# Development and Implementation of an Online Education Program on Advanced Breast Cancer for European Cancer Nurses: ABC4Nurses Project: a Brief Report

**DOI:** 10.1007/s13187-023-02319-3

**Published:** 2023-06-19

**Authors:** Maura Dowling, Amanda Shewbridge, Claire Ryan, Caroline Clancy, Elizabeth Meade, Sarah Sheehan, Celia Díez De Los Rios de La Serna, Gülcan Bağçivan, Grigorios Kotronoulas, Sema Erdem, Violet Aroyo, Bhaveet Radia, Theresa Wiseman, Amanda Drury

**Affiliations:** 1 School of Nursing and Midwifery, University of Galway, Galway, Ireland; 2 Breast Cancer Now, London, UK; 3 Oncology Centre, Maidstone and Tunbridge Wells NHS Trust, Maidstone, UK; 4 Letterkenny University Hospital, Donegal, Ireland; 5 Midland Regional Hospital, Tullamore Offaly, Ireland; 6 School of Nursing, Psychotherapy and Community Health, Dublin City University, Glasnevin, Dublin, 9 Ireland; 7 School of Nursing, Universitat de Barcelona, Barcelona, Spain; 8 School of Nursing, Koc University, Istanbul, Türkiye; 9 School of Medicine, Dentistry and Nursing, University of Glasgow, Glasgow, UK; 10 Europa Donna, Istanbul, Türkiye; 11 Guy’s Cancer Academy, London, UK; 12 The Royal Marsden NHS Foundation Trust, London, UK

**Keywords:** Breast cancer, Metastatic, Curriculum, Nurses, Kirkpatrick

## Abstract

Breast cancer is now the most commonly diagnosed cancer worldwide. Approximately 30% of those who present with early breast cancer later develop advanced breast cancer (ABC). Additionally, approximately 6% have advanced breast cancer at diagnosis. New treatment options result in an extended lifespan dominated by cycles of deterioration and stable disease. Specialist nurse knowledge is key to multidisciplinary care of people with ABC; however, access to education on ABC for nurses is not universally available in Europe. This paper describes the development and implementation of an online bespoke program on ABC care for specialist and generalist nurses in Europe. The project team is affiliated with the European Oncology Nurses Society (EONS) and comprises specialist breast cancer nurses, oncology nurse academics and breast cancer advocates associated with EUROPA DONNA Turkey, an independent non-profit European breast cancer organisation. The program development involved (1) a systematic review of ABC educational resources for cancer nurses; (2) a modified four-round Delphi study to seek agreement on curriculum content and (3) curriculum development, conversion to an interactive online platform and translation into four European languages. The program evaluation will be guided by Kirkpatrick’s framework. The phases described in this short report could guide others involved in developing bespoke cancer education programs.

## Introduction

Breast cancer is now the most commonly diagnosed cancer in Europe accounting for 13.3% of all new cancer cases diagnosed in EU-27 countries with an estimated 355,457 new cases in 2020 [1]. This number will rise following delayed diagnosis and treatment due to COVID-19. People with advanced breast cancer (ABC) have higher unmet needs in terms of distress, depression and anxiety, employment, finances, information and support [2]. Nurses with specialist knowledge and skills are integral to multidisciplinary care; however, most people with ABC do not have access to specialised care [3], and there are wide variations internationally in support provision [4]. Moreover, access to education for cancer nurses is not available in some European countries [5].

In an effort to address this deficiency, a group of specialist breast cancer nurses, oncology nurse academics and experts by experience affiliated with the European Oncology Nursing Society (EONS) commenced work in 2020 to develop ABC4Nurses. ABC4Nurses is a bespoke online advanced breast cancer curriculum for European nurses, translated into four languages (English, Spanish, Turkish and Czech).

Public, patient involvement (PPI) was central to all aspects of this project [6]. We were cognizant of the absence of people living with advanced breast cancer in previous ABC education programs [7] and criticisms that public and patient involvement in health care education is often not meaningful [8]. Therefore, two patient research partners (experts by experience), affiliated with EUROPA DONNA Turkey (https://www.Europa Donna Turkey.org/), contributed throughout the project from protocol development and funding application to program implementation, evaluation, analysis and dissemination. EUROPA DONNA Turkey is an independent non-profit European breast cancer organisation focused on raising awareness of breast cancer and advocating for improved breast cancer education, appropriate screening, optimal treatment and increased research funding.

## Methods

### Phase One: Systematic Review of Advanced Breast Cancer Education Programs for Nurses

While there are published educational standards and competencies for nursing related to ABC [9, 10], synthesising evidence on the availability, scope and outcomes of educational programs related to ABC for nurses was new [7]. Nine peer-reviewed articles and two grey literature reports were included (all from high-income countries), and all offered programs in a face-to-face format, highlighting geographical bias in available education for cancer nurses [7]. We found limited educational programs on ABC for nurses.

### Phase Two: a Modified Four-Round Delphi Study

The systematic review also identified 34 topics related to ABC and nurse competencies. This content guided our Delphi study and development of a Delphi questionnaire. The Delphi technique is based on a series of ‘rounds’ where the views of experts are sought on a series of questions in each round, resulting in an evolving study over time and is commonly used in ascertaining cancer education needs [11].

The Delphi questionnaire was piloted with an expert panel of ten clinical and academic experts on breast cancer nursing, advocacy and education. Following this, 31 experts participated in rounds 1–3 of the Delphi study, and 156 people participated in round 4. Round 4 included people living with ABC (*n* = 72, 46%), healthcare professionals (*n* = 46, 29%), family members or caregivers of a person diagnosed with ABC (*n* = 30, 19%) and advocacy professionals working in the area of ABC (*n* = 8, 5%). In round 4, 36 topics and five of six learning methods reached a consensus. Consensus for the Delphi was defined by at least 80% agreement on the highest three points on a 9-point Likert scale. While the systematic review [7] informed the decision to have six modules, the learning outcomes of the six modules were finalised following the Delphi study [6].

### Phase Three: Module Development and Implementation

The ABC4Nurses curriculum comprises six modules (Table 1), which are accessed via Moodle (a virtual learning platform). Each module requires 20–21 h of study. Following agreement on the learning outcomes, writing of the first module drafts commenced, involving six expert writers (advanced nurse practitioners in oncology, consultant breast nurses and cancer nursing educators), three based in the UK and three in Ireland. The writing process was iterative with many rounds of editing and rewriting to ensure the information provided was broadly applicable across different European oncology healthcare settings. The editing process also involved ensuring the language used would easily accommodate translation from English to three European languages (Spanish, Turkish and Czech) as well as avoiding the use of specific drug names in favour of group labels (for instance, ‘anti-hormone medicines’). When instances of doubt arose on module content that would translate or be applicable beyond the UK or Ireland (for instance, communication acronyms), members of the larger project team based in Spain and Türkiye provided guidance.

**Table 1:** ABC4Nurses modules and learning outcomes

Modules	Learning outcomes
Module 1: understanding metastatic breast cancer	• Understand terminology relevant to advanced breast cancer • Describe signs and symptoms of advanced breast cancer • Explain staging and grading in breast cancer • Explain the incidence and risk factors for metastatic breast cancer • Describe the molecular heterogeneity of metastatic breast cancer • Explain the metastatic cascade in breast cancer
Module 2: supportive care skills in metastatic breast cancer	• Explain what is meant by person-centred supportive care in advanced breast cancer • Explain the nurse’s role in supportive care in advanced breast cancer • Outline how nurses can provide culturally sensitive care in advanced breast cancer • Identify support services for family members and informal caregivers of people living with advanced breast cancer • Describe the communication challenges that arise throughout the metastatic breast cancer trajectory (information provision, living with uncertainty, disease progression, transition to palliative care, end-of-life care) • Outline barriers to therapeutic communication in advanced breast cancer • Outline the six-step protocol used in SPIKES, when giving bad news • Outline communication challenges with family members and informal caregivers of people with cancer • Describe how to de-escalate conflict situations and strategies to establish a working relationship with a dominant family member • Understand coping with emotional demands of caring for people with advanced breast cancer (family, informal carers and members of the MDT)
Module 3: treatment options and decision-making in metastatic breast cancer	• Explain what is meant by inter-disciplinary/multi-disciplinary/collaborative care • Explain the nurse’s role within the advanced breast cancer multidisciplinary team • Explain international consensus guidelines for advanced breast cancer and the role of specialised breast cancer units • Describe the objectives of treatment in advanced breast cancer • Describe the factors that influence cancer treatment choices in advanced breast cancer • Outline the factors underpinning informed decision-making in advanced breast cancer • Explain the role of chemotherapy in the management of advanced breast cancer. • Explain the role of targeted therapy in the management of advanced breast cancer • Explain the role of hormone therapy in the management of advanced breast cancer • Explain the role of immunotherapy in the management of advanced breast cancer • Explain the role of radiotherapy in the management of advanced breast cancer symptoms and acute care needs • Describe the role of clinical trials in advanced breast cancer
Module 4: acute care needs of metastatic breast cancer	• Explain the common terminology criteria for adverse events (CTCAE) • Outline the most common and most dangerous side effects of treatment in advanced breast cancer • Describe pathophysiology and management of hypercalcemia in malignancy • Describe the pathophysiology and management of malignant spinal cord compression • Describe the pathophysiology and management of neutropenic sepsis • Describe the pathophysiology of disease and treatment-related thromboembolism • Describe the pathophysiology and management of pleural effusion • Describe the pathophysiology and management of superior vena cava obstruction • Describe pathophysiology and management of brain metastases
Module 5: assessing and managing common symptoms in metastatic breast cancer	• Explain person-centred assessment in advanced breast cancer • Explain what is meant by a symptom cluster • Describe the symptoms of the ‘fatigue, depression, sleep-wake disturbance’ symptom cluster and evidence-based interventions • Describe the role of bone modifiers (bisphosphonates and denosumab) in the management of metastatic bone pain and reducing skeletal-related events • Describe symptom management for fungating breast wounds to reduce pain, bleeding, discharge and offensive odour • Describe symptom management for disease-related breathlessness • Describe symptom management for disease and treatment-related gastrointestinal symptoms
Module 6: living well with metastatic breast cancer	• Outline the physical, psychological, social and spiritual implications of living with metastatic breast cancer • Outline the impact of a metastatic breast cancer diagnosis on family, children and informal caregivers • Explain the meaning of living well for individuals with advanced breast cancer • Identify support services for people living with advanced breast cancer • Describe interventions that support the self-management skills of people living with advanced breast cancer • Outline the role of complementary therapies in symptom management and wellbeing in advanced breast cancer • Explain the factors necessary for integrating early palliative care to support wellbeing throughout the treatment journey in metastatic breast cancer • Describe the role of advance care planning in end-of-life communication in metastatic breast cancer

The involvement of a learning designer was integral to program development. Learning designers bring skills in learning theory, curriculum development and course design [12]. As each module was completed, two of the expert writing team worked closely with the learning designer who, using the written content, developed an interactive learning experience using discussion activities, case studies, animation, bespoke graphics and interactive exercises. This was developed using Articulate Rise ©. This process took 4 months with numerous iterations of each module.

Funding for the project was obtained following a grant application process. The funding was used to meet the costs of the learning designer, the Moodle platform and administrative support to maintain the platform registrations and graduations. In addition, the funding provided honoraria for the expert writers and academic leads.

## Discussion

Methods of teaching and learning to support the delivery of the proposed online education program were integrated into the curriculum. Teaching and learning methods include quizzes, video clips, problem solving/problem-based learning, caregiver perspectives and experiences of care, and direction to additional educational resources. Three case studies are also utilised to act as an ‘anchor’ for student exercises. Participants are introduced to persons living with ABC in the first module and provided with further details of the person’s life and challenges throughout each module. For instance, the case study of ‘Elizabeta’ focuses on a diagnosis of grade 3 invasive ductal carcinoma, ER+ve, PR+ve, HER2-ve, with a large tumour infiltrating the chest wall, bilateral axillary masses and nodes in the supraclavicular fossa, small pleural effusions and bone metastases in the spine and right femur. ‘Elizabeta’ cares for her husband who has dementia and worries about who will care him while she tries to manage hospital appointments and manage treatment side effects.

Phase four of the project, program evaluation is anticipated to take 6 months and includes records of nurses registering in each language, numbers active on the learning platform monthly, numbers completing the program and the number of inactive participants following registration. The evaluation was developed in tandem with the writing of the module content so that pre-study and post-study knowledge questions asked within each module would also inform the pre- and post-program evaluation and minimise respondent burden. Therefore, ethical approval for the program evaluation was sought during the final phase of the module development.

The program evaluation will utilise a mixed-method approach using the four levels of the Kirkpatrick evaluation framework [13] (1. reaction; 2. learning; 3. behaviour change; 4. results). This framework is the most widely cited in educational evaluations and has been used extensively in evaluating cancer education [14]. To measure levels 1 and 2 of the Kirkpatrick framework, program participants will be asked to complete a questionnaire assessing their knowledge and skills and personal views before and after each of the six modules. Questions included were adopted from previously used cancer education evaluations and also based on the module content. For instance, participants will be asked to respond on a numerical scale of 1 (not at all prepared) to 10 (very well prepared), how well prepared they think they are to care for people with cancer [15].

Levels 3 and 4 of the framework are more challenging to capture, and Kirkpatrick’s framework does not provide clear guidance on how to identify if participants’ added knowledge, skills or awareness has made an impact on their practice. However, it is acknowledged that finding a causal link between an educational intervention and changes in clinical outcomes is problematic [14]. In an effort to also include levels 3 and 4 of Kirkpatrick’s framework, participants will complete a questionnaire at the end of the program on their experiences and satisfaction with the program and will be invited to participate in qualitative interviews via Zoom in their own language of preference (English, Turkish, Spanish or Czech). Participants will also be asked to share information about the program with their line manager and explore their line manager’s willingness to be interviewed about their views on the clinical impact of the program.

In conclusion, the development and implementation of ABC4Nurses will address a gap in the educational needs of generalist and specialist nurses in Europe and may improve the service provided by nurses to people living with ABC. The process we have described will provide guidance to others in the development of online education programs for cancer healthcare professionals. Central to the success of this program development was the establishment of a team with different skills and experiences in cancer care and curriculum design. Also, the involvement of breast cancer advocates and the translation of participant information materials and advertisement in round 4 were fundamental to the high response rate from people living with breast cancer in the modified Delphi questionnaire. Through their contribution, we know what people with ABC want nurses to know and what skills they need.
